# Prevalence and associated factors of hypertension among adults in Ethiopia: a community based cross-sectional study

**DOI:** 10.1186/s13104-017-2966-1

**Published:** 2017-11-28

**Authors:** Henok Asresahegn, Frew Tadesse, Ermias Beyene

**Affiliations:** 1 School of Public Health, College of Health Sciences and Medicine, Jigjiga University, Jigjiga, Ethiopia; 2 School of Medicine, College of Health Sciences and Medicine, Jigjiga University, Jigjiga, Ethiopia

**Keywords:** Hypertension, Prevalence, Determinants, Obesity, Ethiopia

## Abstract

**Background:**

Hypertension is a growing public health problem in many developing countries including Ethiopia. It is a silent killer and most patients are detected to have it incidentally when they are admitted to hospital for unrelated disease or subjected to pre-employment or preoperative medical checkups. Information on the prevalence of hypertension and its associated factors is to be considered vital to focus and improve prevention and control of cardiovascular diseases. The study design was a cross-sectional survey. The study population consisted of adults aged 25–65 years who lived in Jigjiga city of eastern Ethiopia for at least 6 months. Data were collected using a pretested structured questionnaire, and blood pressure was measured using standardized instruments by trained clinical nurses. Hypertension was defined as having Systolic BP ≥ 140 mmHg or Diastolic BP ≥ 90 mmHg or reported use of regular anti-hypertensive medications prescribed by professionals for raised BP. Data were entered into a computer using Epi Info Version 3.5.1 and exported to SPSS version 16.0 for analysis. Multiple logistic regressions were fitted and Odds ratios with 95% confidence intervals were calculated to identify independently associated factors.

**Results:**

The prevalence of hypertension was 28.3%. Family history of Hypertension [Adjusted OR 5.7; 95% CI (2.9, 10.9)], having high level of income [Adjusted OR 3.1; 95% CI (1.5, 6.3)], being male [Adjusted OR 2.4; 95% CI (1.3, 4.3)], being above grade 12 [Adjusted OR 2.2; 95% CI (1.2, 3.9)], and having BMI ≥ 25 [Adjusted OR 2.0; 95% CI (1.1, 3.5)] were significantly associated with hypertension.

**Conclusion:**

Consistent with the literatures, the prevalence of hypertension was high and may show a hidden epidemic in this population. If established with more robust and nationally representative studies, the finding calls for efficient health screening and regular checkups as well as interventions promoting healthy lifestyles. Accordingly, health promotion regarding hypertension should be provided in the population as means of primary prevention.

**Electronic supplementary material:**

The online version of this article (10.1186/s13104-017-2966-1) contains supplementary material, which is available to authorized users.

## Background

Hypertension is a state of elevated systemic blood pressure which is commonly asymptomatic. It is a major cardiovascular risk factor that is closely associated with lethal complications like coronary artery disease, cerebro-vascular accidents, heart and renal failure [1]. Hypertension is an overwhelming global challenge, which ranks third as a means of reduction in disability-adjusted life-years [2]. Besides, it is the leading cause of mortality [3]. Globally, nearly one billion people have hypertension; of these, two-thirds are in developing countries [4]. The burden of chronic non-communicable diseases (NCDs) in developing countries has risen sharply in recent years. The new epidemic of hypertension and cardio-vascular diseases is not only an important public health problem, but it will also have a big economic impact as a significant proportion of the productive population becomes chronically ill or die, leaving their families in poverty [5].

Hypertension is a silent killer and most patients are detected to have it incidentally when they are admitted to hospital for unrelated disease or subjected to pre-employment or preoperative medical checkups. The exact causes of high blood pressure are not known, but several factors and conditions may play a role in its development [1]. Few studies indicate that the disease has become significant public health problem especially in the major cities of Ethiopia. According to the studies conducted in Addis Ababa and Gondar the prevalence of hypertension was high probably indicating a hidden epidemic in those communities [6, 7]. To our knowledge, there have not been any published studies in this regard in Somali region of Ethiopia. Hence, we tried to find the prevalence and associated factors of high blood pressure among adults in the region. The findings of this study will be useful to raise awareness among policy makers and the public at large, about the magnitude of high blood pressure and related risk factors of cardiovascular diseases, and thereby, contribute to the design and implementation of appropriate interventions.

## Methods

### Study population

A cross-sectional study was conducted among randomly selected adults aged 25–65 years in Jigjiga city of Somali Regional State in Ethiopia from October to November 2014. The Study participants were permanent residents of Jigjiga city who have been living there at least for 6 months. Subjects with obvious physical deformity, pregnant women and known psychiatric patients who were taking medication were excluded from the study. Single population proportion formula was applied to calculate the sample size by using hypertension prevalence rate of 28.3% [6], with 5% precision at 95% confidence level, 5% non-response rate, and design effect of 1.5 which as a result give a total sample size of 492.

This study was conducted in accordance with the STEP-wise approach of the World Health Organization (WHO) for NCD surveillance in developing countries [8]. The approach has three levels: (1) questionnaire to gather demographic and behavioral information, (2) simple physical measurements, and (3) biochemical tests. The present study used the first two steps; questionnaire survey, and anthropometric and blood pressure measurement. Consumption patterns of the participants for various food groups were studied using a Food Frequency Questionnaire.

### Data collection and variable specification

Participants were interviewed by trained interviewers using the WHO STEPS-structured questionnaire. In accordance with the STEPS manual, questions related to alcohol and substance use were tailored to reflect the local context of Ethiopia. The questionnaire was first written in English, translated into local languages (*Amharic* and *Somaligna)* by experts and translated back into English by a panel of professionals who speak both languages. The questionnaire was pretested before the initiation of the study and contained information regarding socio-demographic characteristics, tobacco and alcohol use, and nutritional status. A 3-days training of the contents of the STEPS questionnaire, data collection techniques, and ethical conduct of human research was provided to research interviewers prior to the commencement of the study.

Blood pressure (BP) was measured using a digital measuring device with participants sitting after resting for at least 5 min. Two BP measurements were taken with at least 3 min intervals between consecutive measurements. The mean systolic and diastolic BP from the first and second measurement was analyzed. The measurement was taken with each subject sitting on a chair and supported hand. The measurement was taken early in the morning from 7 a.m. to 10 a.m. and in the afternoon after 4 p.m. in a calm environment. Hypertension was defined as mean systolic blood pressure (SBP) ≥ 140 mmHg or mean diastolic blood pressure (DBP) ≥ 90 mmHg. The study protocol was approved by the research and publication technology transfer office of Jigjiga University.

### Statistical analysis

Data were entered into EPI INFO (Version 3.5.1), and exported to SPSS (Version 16.0) for statistical analysis. We first explored frequency distributions of socio demographical and behavioral characteristics of subjects and descriptive statistics was used to summarize and present the information in the form of mean, median, percentages and tables with 95% confidence intervals for prevalence estimates. A binary logistic regression model was used to examine factors associated with Hypertension among adults (0 = Non-Hypertensive, 1 = Hypertensive). Variables which showed association with dependent variable in the bivariate analyses at 0.2 were entered into multivariate logistic regression model. Multiple binary logistic regression analysis was used to examine the association between variables and hypertension adjusting for other potential confounders. A *p* value of less than 0.05 was used to define statistical significance. Both crude and adjusted odds ratio are presented with a 95% confidence interval. The Hosmer–Lemeshow goodness-of-fit and Omnibus tests of model coefficients tests with enter procedure were used to test for model fitness. The explanatory variables were tested for multi-collinearity before entering them into the multivariable model, using the variance inflation factor (VIF) test, the Tolerance test, and values of the standard error. Body mass index (BMI) was computed using weight (Kg) per height (m)^2^. Concerning to income, those with monthly income of < 1970 birr, 1970–2999 birr, and ≥ 3000 birr were labeled as low level, medium level and high level of income respectively.

Physical activity was classified according to the STEPS manual [8] as follows;

*High*

- Vigorous-intensity activity on at least 3 days achieving a minimum total physical activity of at least 1500 min/week OR
- 7 or more days of any combination of walking, moderate-intensity or vigorous intensity activities achieving a minimum Total physical activity of at least 3000 min/week [28]

*Moderate*

- 3 or more days of vigorous-intensity activity of at least 20 min per day OR
- 5 or more days of moderate-intensity activity and/or walking of at least 30 min per day OR
- 5 or more days of any combination of walking, moderate-intensity **or** vigorous intensity activities achieving a minimum total physical activity of at least 600 min/week.

*Low* is the lowest level of physical activity. Those individuals who do not meet the criteria for moderate and high are considered.

Smoking status was defined as follows;

Current smoker: Someone who has smoked greater than 100 cigarettes in their life time and has smoked in the last 28 days.
Previous smoker: Someone who has smoked greater than 100 cigarettes in their life time but hasn’t smoked in the last 28 days.
Non-smoker: Someone who hasn’t smoked greater than 100 cigarettes in their life time and doesn’t currently smoke.

## Results

### Study demographics

A total of 487 adults provided data for analysis. The mean and median ages of the study participants were 35 and 32 years old respectively (Table 1). Regarding the self or family history of any chronic disease; 50 (10.3%), and 16 (3.3%) of the total study participants were known hypertensive, and diabetes mellitus (DM) patients respectively, while 82 (16.8%) and 64 (13.1%) have family history of hypertension and DM respectively. On the other hand, 182 (37.4%) and 131 (26.9%) of the total respondents were Chat chewer and smoker respectively (Table 1).

**Table 1:** Socio-demographic characteristics of study participants in Jigjiga city, October–November 2014

Variables	Frequency	Percent
Sex		
Male	2381	48.9
Female	249	51.1
Age (years) [Mean = 35]		
25–34	285	58.5
35–44	125	25.7
45–54	48	9.9
55–65	29	6.0
Marital status		
Single	176	36.1
Married	254	52.2
Others	57	11.7
Highest level of education		
Illiterate	9	1.8
Literate but no formal education	34	7.0
Primary school (1–8)	104	21.4
Secondary school (9–12)	133	27.3
Certificate or higher	207	42.5
Income (birr)		
Low level	139	33.3
Medium level	157	37.6
High level	121	29.1

### Prevalence of hypertension

Blood pressure measurements were done to all the study subjects to check for hypertension. The mean systolic and diastolic BP results were 125.7 mmHg (± 16 SD) and 79.7 mmHg (± 9.2 SD).The overall prevalence of hypertension was 28.3% (95% CI 24.5, 32.5). Among all hypertensive people identified, 88 (63.8%) did not know they have had hypertension (newly screened). Of the 50 hypertensive people who reported using anti-hypertensive medications during data collection period, 34.0% had normal BP on measurement (Table 2).

**Table 2:** Hypertension prevalence across behavioral and dietary characteristics of respondents in Jigjiga city October–November 2014

Variables	n	Percent	Hypertensive (%)
Positive family history of hypertension			
Yes	82	16.8%	56.1
No	405	83.2%	22.7
Positive family history of DM			
Yes	64	13.1%	31.2
No	423	86.9%	27.9
Known DM patient			
Yes	16	3.3%	68.8
No	471	96.7%	27.0
Smoking history			
Nonsmoker	410	84.2%	26.8
Previous smoker	10	2.1%	20.0
Current smoker	67	13.8%	38.8
Do you drink alcohol?			
Yes	131	26.9%	34.4
No	356	73.1%	26.1
Do you chew chat?			
Yes	182	37.4%	31.9
No	305	62.6%	26.2
Physical activity			
Low	264	58.0	26.9
Medium	124	27.3	27.4
High	67	14.7	31.3
Central obesity			
Non obese	152	31.2	25.0
Obese	335	68.8	29.9
BMI (Kg/m^2^)			
< 25	328	67.4	22.6
≥ 25	159	32.6	40.3
Frequency of eating per day			
≤ 3 times	386	79.3	26.7
> 3 times	101	20.7	34.7
Frequency of eating oil and fatty foods			
Not frequent	296	60.8	25.3
Frequent	191	39.2	33.0
Frequency of eating sugar and sweets			
Not frequent	355	72.9	30.7
Frequent	132	27.1	22.0

### Factors associated with hypertension

Having family history of hypertension, having high level of income, being male, being below grade 12, and having BMI ≥ 25 were significantly associated with hypertension for the overall study participants (Table 3). Among all, those who have family history of Hypertension were nearly six times more likely to be hypertensive when compared to those who haven’t [Adjusted OR 5.7; 95% CI (2.9, 10.9)], those who had high level of income were three times more likely to be hypertensive when compared to those who had low level of income [Adjusted OR 3.1; 95% CI (1.5, 6.3)], those who are male were 2.4 times more likely to be hypertensive when compared to those female participants[Adjusted OR 2.4; 95% CI (1.3, 4.3)], those who were below grade 12 were two times more likely to be hypertensive when compared to those who are above grade 12 [Adjusted OR 2.2; 95% CI (1.2, 3.9)], and those who had BMI ≥ 25 were two times more likely to be hypertensive when compared to those who had BMI < 25 [Adjusted OR 2.0; 95% CI (1.1, 3.5)]. The other variables were not significantly associated with hypertension after adjusting for confounders (Table 3).

**Table 3:** Factors associated with Hypertension among adults in Jigjiga city, October–November 2014

Variables	Status of hypertension, n (%)		COR (95% CI)	AOR (95% CI)
	Hypertensive	Non-hypertensive		
Physical activity				
Low	71 (26.9)	193 (73.1)	0.8 (0.5, 1.4)	0.9 (0.5, 2.0)
Medium	34 (27.4)	90 (72.6)	0.8 (0.4, 1.6)	0.7 (0.3, 1.5)
High	21 (31.3)	46 (68.7)	1	1
Central obesity				
Non obese	38 (25.2)	113 (74.8)	1	1
Obese	99 (29.6)	235 (70.4)	1.3 (0.8, 1.9)	1.0 (0.6, 1.8)
BMI (Kg/m^2^)				
< 25	74 (53.6)	254 (72.8)	1	1
≥ 25	64 (46.4)	95 (27.2)	2.3 (1.5, 3.5)	2.0 (1.1, 3.5)*
Sex				
Male	74 (31.1)	164 (68.9)	1.3 (0.9, 1.9)	2.4 (1.3, 4.3)*
Female	64 (25.7)	185 (74.3)	1	1
Age (years)				
25–34	60 (21.1)	225 (78.9)	1	1
35–44	38 (30.4)	87 (69.6)	1.6 (1.1, 2.6)	0.6 (0.3, 1.2)
45–54	20 (41.7)	28 (58.3)	2.7 (1.4, 5.1)	1.5 (0.6, 3.5)
55–65	20 (69.0)	9 (31.0)	8.3 (3.6, 19.2)	2.1 (0.6, 7.9)
Educational status				
Grade 12 or lower	91 (32.5)	189 (67.5)	1.6 (1.1, 2.5)	2.2 (1.2, 3.9)*
Above grade 12	47 (22.7)	160 (77.3)	1	1
Income (birr)				
Low level	21 (15.1)	118 (84.9)	1	1
Medium level	50 (31.8)	107 (68.2)	2.6 (1.5, 4.7)	1.9 (0.9, 3.7)
High level	46 (38.0)	75 (62.0)	3.4 (1.9, 6.2)	3.1 (1.5, 6.3)*
Family history of Hypertension				
Yes	46 (56.1)	36 (43.9)	4.4 (2.7, 7.1)	5.7 (3.0, 10.9)**
No	92 (22.7)	313 (77.3)	1	1
DM status				
Diabetic	11 (68.8)	5 (31.2)	6.0 (2.0, 17.5)	3.9 (0.9, 17.5)
Non diabetic	127 (27.0)	344 (73.0)	1	1
Frequency of eating deep fries				
Not frequent	46 (35.4)	84 (64.6)	1	1
Frequent	92 (25.8)	265 (74.2)	0.6 (0.4, 0.9)	0.7 (0.3, 1.3)

** Significance level of < 0.001 * Significance level of < 0.05

### Dietary habits of respondents

About 49% of the study subjects eat meat and eggs at least two to four times a week. While 39% of the respondents eat oil and fats at least two to four times a week. Regarding the frequency of consumption of sugars and sweets; 89 (18.3%) eat two to four times per week, and only 43 (9%) eat at least once a day. Majority of the respondents 92.4, 98.2, and 85.2% eat breakfast, lunch, and dinner on daily basis respectively. Concerning to the frequency of eating of deep fries; 327 (67.1%) eat sometimes, 130 (26.7%) had never eaten deep fries, and 30 (6.2%) eat daily. Two hundred forty one (49.5%) had never eaten any visible fat in a meat, while 234 (48.0%) eat any visible fat in a meat sometimes, and the rest 12 (2.5%) eat daily.

### Physical activity

Among the 455 study participants who respond about their physical activity, 264 (58.0%) had low level of physical activity, 124 (27.3%) had moderate level of physical activity, and 67 (14.7%) had high level of physical activity. Thirty two (6.6%) didn’t respond to the physical activity variable.

### Anthropometric measurements

The mean and median heights of the study participants were 1.65 m whereas; the mean and median weights of the study participants were 65.2 and 63 kg respectively. Hence, the mean and median BMI of the study participants were 23.9 and 23.3 kg/m^2^ old respectively. Furthermore, the mean and median waist circumferences of the study participants were 82.1 and 80 cm respectively, while the mean and median hip circumferences of the study participants were 90.6 and 91 cm respectively. Therefore, the mean and median waist-to-hip ratio (WHR) of the study participants was 0.92, and 0.90 respectively. Regarding the centrally obesity of the study participants, 335 (68.8%) were centrally obese.

## Discussion

This study provides information regarding the prevalence and associated factors of hypertension among adults in living in the Semi-pastoralist community of eastern Ethiopia. It has demonstrated a 28.3% prevalence of hypertension. The present study depicts that having family history of Hypertension, having high level of income, being male, being above grade 12, and having BMI ≥ 25 were significantly associated with hypertension.

The prevalence of hypertension by self-report and physical measurement was 10.3 and 24.9%, respectively. More than twofold difference between the two measures indicates that a significant number of the population was not aware of their hypertension status which calls for appropriate and timely intervention. It is known that most individuals with high blood pressure do not have symptoms until complication arises to result in sudden death from heart attack or sudden intracranial bleeding or developed severe disability such as stroke as well as heart failure.

The overall prevalence of hypertension in our study was 28.3% which is consistent with the most recent community-based studies in Ethiopia and Zambia (28.3% in Gondar city, and 30% in Addis Ababa, and 31.8% in *Zambia*) [6, 7, 9]. However it is significantly higher than the findings of the study done in south west Ethiopia (13.2%), and slightly higher than that of Jimma, Ethiopia (21.3%), Uganda (22%) and Tanzania 23.7% [10–13]. This could partly be due to the fact that, this study was conducted only in an urban setting, whereas the study conducted in south west Ethiopia was conducted in both urban and rural settings as well as the age difference in the study populations, and that of Uganda and Tanzania were conducted in rural settings.

In our study, individuals with positive family history of hypertension were more likely to be hypertensive. In agreement with this, many other previous studies [14–16] reported that family history of hypertension was significantly associated with being hypertensive in this study. This could be explained by the fact that genetic factors account for one-third to one-half of the risk of hypertension. Blood relatives tend to have many of the same *genes* that can predispose a person to high blood pressure, heart disease, or stroke [17, 18]. Moreover, the tendency of individuals to follow risky life styles of their family could have also played a role for this association.

This study showed highest risk of hypertension among subjects who have high level of income. Some studies showed that the income distributions and hypertension were nonlinear indicating elevated levels in low income as well as in high income groups [19–21]. In the present study, being male was more likely to be hypertensive. This finding is in line with previous research reports [7, 22–24]. Participants who were below grade 12 were more likely to be hypertensive in this study. However, this study tried to see educational status by re-categorizing to above grade 4 and below 4 or above grade 8 and below grade 8, but there was no association at all. This finding is consistent with the results from previous studies [25–27] that reported the positive relationship between hypertension and low education. Such association may be due to lack of awareness of risk factors of hypertension [28] as a result of the low educational status.

Participants with a BMI ≥ 24.9 were more likely to be hypertensive compared to those with a BMI of 24.9 or lower, which is consistent with multiple previous studies that have reported a strong association between hypertension and BMI [6, 22, 29–31]. Obesity is a well-established risk factor for hypertension [32–35]. Frequently used measures of obesity are BMI, skin fold thickness, estimation of per cent body fat and body weight and hip-waist ratio [36]. Obesity has been recognized as the most important risk factor for developing hypertension. Several epidemiological studies from different populations have reported a significant association between obesity and hypertension [23, 37–41]. The link between obesity and hypertension is through neuro-endocrine mechanisms and most recently, factors derived from adipose tissue are thought to play a major role.

The study has a number of strengths including being a community based study which can truly describe the general population in contrast to the many reports that have emanated from hospital based studies. The main limitation of this study is that it is a simple cross-sectional, and didn’t include variables such as psychological stress and biochemical measurements. Hence other analytic studies that incorporate biochemical measurements should be carried out in order to assure the temporal relationship between hypertension and the explanatory variables. Moreover, there is a need to carry out research on the reasons for difference in prevalence of hypertension among male and female.

## Conclusions

Findings of this study indicate that hypertension has become an important public health problem among adults in jigjiga city, with a huge discrepancy in the proportion of those who were newly screened and those who are known hypertensive subjects. This may indicate a hidden epidemic that underscores the need for comprehensive evaluation of prevalence of hypertension and other cardiovascular diseases in this population. If established with more robust and nationally representative studies, the finding calls for efficient health screening and regular checkups as well as interventions promoting healthy lifestyles. Certain factors like family history of hypertension, having high level of income, being male, being above grade 12, and having BMI ≥ 25 were found to be associated with high BP. Therefore, community based screening programs for Hypertension and its risk factors need to be carried out. Especially there is a need for routine screening for hypertension for those overweight or obese, low level educational status, those with positive family history of hypertension and high level of income, as they have an increased likelihood of developing hypertension.

## Additional files

**Additional file 1.** Hypertension data set. SPSS data set for the research entitled “Prevalence and associated factors of hypertension among adults in Ethiopia: a community based cross-sectional study”.

**Additional file 2.** Questionnaire hypertension BMC RN. Study tool for the research entitled “Prevalence and associated factors of hypertension among adults in Ethiopia: a community based cross-sectional study”.

## Abbreviations

BMI: body mass index
BP: blood pressure
CHD: coronary heart disease
DBP: diastolic blood pressure
DM: diabetes mellitus
NCD: non-communicable diseases
SBP: systolic blood pressure
SRS: simple random sampling
WHO: World Health Organization
WHR: waist-to-hip ratio
