# Supplementary material for: Prevalence and associated factors of hypertension among adults in Ethiopia: a community based cross-sectional study
Source: BMC Res Notes. 2017 Nov 28;10:629. doi: 10.1186/s13104-017-2966-1 (PMC5704552; doi:10.1186/s13104-017-2966-1)
Supplement: Supplementary file 2 — Additional file 2. Questionnaire hypertension BMC RN. Study tool for the research entitled “Prevalence and associated factors of hypertension among adults in Ethiopia: a community based cross-sectional study”. [file 13104_2017_2966_MOESM2_ESM.docx]

**JIGJIGA UNIVERSITY**

**COLLEGE OF HEALTH SCIENCES**

**Survey Questionnaire to Assess Prevalence and associated factors of hypertension among adults in Ethiopia**

INFORMATION SHEET

I am ----------. We came from Jigjiga University, Collage of Health Science, department of Public Health to conduct a research on comparison of measures of adiposity and hypertension among adults in Jigjiga city. The aim of the study is to assess the burden of overweight/obesity and hypertension. Therefore, this study will have a great contribution in the control and prevention of hypertension and obesity and related chronic diseases. Besides, we believe that this study will help in attracting governmental and non-governmental organizations and contribute their part on these problems.

During the study period height, weight, waist circumference, and blood pressure will be measured using standardized instruments. Only light clothes will be wearing during measurement of body weight and Height will be measured using measuring board with bare foot. You will also be interviewed about your feeding practice, physical activity and health habits. This study does not bring any harm to your health. If you feel discomfort with the questions, please feel free to drop at any time you want. This process will take about 30 minutes.

Finally, what we want to assure you is that your name and address will not be mentioned and handed over to others. However, the result will be organized and documented and might be submitted/given to the concerned Health Organizations or other bodies.

Investigator Address:

Henok Asresahegn Asfaw

Tel: +251-911-57-11-39

Email: hasresahegn01@gmail.com

Frew Taddesse

<Tel:-> +251-913-78-24-29

Email:-fretaabrish@gmail.com

Dr. Ermias Bayene

Tel: +251-911-00 45 52

**Consent form**

Having the above information, I invite you to participate in the study.

I the under signed, would like to confirm that, as I give consent to participate in the study, it is with clear understanding and recognition of:

1. The objective of the study
2. My right to resign from the study during any stage of the study

I confirmed my agreement with my signature after the detailed objective of the study has been explained to me in the language I understand well.

Signature (participant’s) ____________ Signature (collectors) _________________

Date ______________________ Date _____________________

| **No.** | **Identifications** | **Response** |
| --- | --- | --- |
| 1 | Questionnaire number |  |
| 2 | Region |  |
| 3 | Zone |  |
| 4 | City |  |
| 5 | Kebele |  |
| 6 | House number |  |
| 7 | Name of interviewer |  |
| 8 | Name of supervisor |  |
| 9 | Date (dd/mm/yy) | /_____ /_____ /2007 EC |

**Section I – Socio-demographic characteristics of respondent**

| No | Questions | Choice |
| --- | --- | --- |
| 1.1 | Age of the respondent | ______________years |
| 1.2 | Sex of the respondent | 1. Male 2. Female |
| 1.3 | What is the highest level of school or grade you have completed? | 1. Unable to read or write 2. Can only read and write 3. Primary Cycle 1 (1-4) 4. Primary Cycle 2 (5-8) 5. Secondary (9-12) 6. TVET(10+1 or 10+2) 7. TVET(10+3 /Diploma 8. University Degree or Above 9. Other (specify)__________________ |
| 1.4 | What is your Religion? | 1. Muslim 2. Orthodox 3. Catholic 4. Protestant 5. Other( specify)--------- |
| 1.5 | What is your current marital status? | 1. Single 2. Married 3. Widowed 4. Separated 5. Divorced |
| 1.6 | To which ethnic group do you belong? | 1. Oromo 2. Amhara 3. Guraghie 4. Somali 5. Hadiya 6. Tigre 7. Other(specify) ________ |
| 1.7 | For how many years have you lived in this city? | ___________ |
| 1.8 | What is your current main work/occupation? | 1. Farmer 2. Housewife 3. Daily labourer 4. Government employee 5. NGO employee 6. Trader 7. Student 8. Other (specify)_______ |
| 1.9 | Average monthly income | ______________birr |
| 1.10 | Are you hypertensive? | 1. Yes 2. No |
| 1.11 | Do you have family history of hypertension? | 1. Yes 2. No |
| 1.12 | Are you diabetic? | 1. Yes 2. No |
| 1.13 | Do you have family history of DM? | 1. Yes 2. No |
| 1.14 | Smoking habit | 1. Current smoker 2. Non smoker 3. Pervious smoker |
| 1.15 | How often do you smoke? | 1. Less than once a month 2. Once to three times per month 3. Once a week 4. Two to four times per week 5. Daily |
| 1.16 | Alcohol drinking | 1. Yes 2. No |
| 1.17 | How often do you drink alcohol? | 1. Less than once a month 2. Once to three times per month 3. Once a week 4. Two to four times per week 5. Daily |
| 1.18 | Do you chew chat? | 1. Yes 2. No |
| 1.19 | How often do you chew chat? | 1. Less than once a month 2. Once to three times per month 3. Once a week 4. Two to four times per week 5. Daily |

**Section II: NUTRITION AND DIET INFORMATION**

| As you answer these questions please think of a typical month in the last year. | | |
| --- | --- | --- |
| No | Questions | Choices |
| 2.1 | How often do you eat Bread and Cereals? | 1. Never 2. Less than once a month 3. Once to three times per month 4. Once a week 5. Two to four times per week 6. Ones per day 7. More than twice per day |
| 2.2 | How often do you eat Greens and vegetables? | 1. Never 2. Less than once a month 3. Once to three times per month 4. Once a week 5. Two to four times per week 6. Ones per day 7. More than twice per day |
| 2.3 | How often do you eat Fruits? | 1. Never 2. Less than once a month 3. Once to three times per month 4. Once a week 5. Two to four times per week 6. Ones per day 7. More than twice per day |
| 2.4 | How often do you eat Milk, cheeses, yogurt? | 1. Never 2. Less than once a month 3. Once to three times per month 4. Once a week 5. Two to four times per week 6. Ones per day 7. More than twice per day |
| 2.5 | How often do you eat Meat and eggs? | 1. Never 2. Less than once a month 3. Once to three times per month 4. Once a week 5. Two to four times per week 6. Ones per day 7. More than twice per day |
| 2.6 | How often do you eat Beans? | 1. Never 2. Less than once a month 3. Once to three times per month 4. Once a week 5. Two to four times per week 6. Ones per day 7. More than twice per day |
| 2.7 | How often do you eat Oil and fat? | 1. Never 2. Less than once a month 3. Once to three times per month 4. Once a week 5. Two to four times per week 6. Ones per day 7. More than twice per day |
| 2.8 | How often do you eat Sugar and sweets? | 1. Never 2. Less than once a month 3. Once to three times per month 4. Once a week 5. Two to four times per week 6. Ones per day 7. More than twice per day |

**Section III: EATING AND HEALTH HABITS**

| The next questions ask about your dieting habit for the last one year. | | | |
| --- | --- | --- | --- |
| **No** | **Questions** | **Response** | **Coding** |
| 3.0 | How many times you normally eat per day? | 1. <1 meal a day 2. 1 meal a day 3. 2 meals a day 4. 3 meals a day 5. >3 meals a day |  |
| 3.1 | How often do you eat Breakfast? | 1. Daily 2. Sometimes 3. Never |  |
| 3.3 | How often do you eat during Tea time? | 1. Daily 2. Sometimes 3. Never |  |
| 3.4 | How often do you eat Lunch? | 1. Daily 2. Sometimes 3. Never |  |
| 3.5 | How often do you eat Dinner? | 1. Daily 2. Sometimes 3. Never |  |
| 3.6 | How often do you eat during Bed time? | 1. Daily 2. Sometimes 3. Never |  |
| 3.7 | How often do you eat fast foods? | 1. Daily 2. Sometimes 3. Never |  |
| 3.8 | How often do you eat deep fries? | 1. Daily 2. Sometimes 3. Never |  |
| 3.9 | How often do you eat visible fat in meat? | 1. Daily 2. Sometimes 3. Never |  |
| 3.10 | How many hours do you spent with TV programs, video games or computer | 1. <30min a day 2. 30-59min a day 3. 60-89min a day 4. ≥90 minutes a day |  |
| 3.11 | For how many hours do you sleep. | 1. <6 hrs a day 2. 6-9hrs a day 3. ≥10hrs a day |  |
| 3.12 | Which one do you usually use to go from place to place? | 1. Walking 2. Cycle 3. Driven |  |

**Section IV: PHYSICAL ACTIVITY QUESTIONNAIRE**

| **Physical activity Questionnaire**  **Below are questions about individual’s physical activity levels. Please read the descriptions and answer the questions even if you do not consider yourself to be an active person. Consider all activities, those you do at school, as part of your house and yard work, to get from place to place, and in your spare time for recreation, exercise or sport.** | | | | | |
| --- | --- | --- | --- | --- | --- |
|  | ***Hard physical activity:***  ***Think about all the vigorous activities which take hard physical effort that you did in the last 7 days. Vigorous activities make your breath harder than normal and may include heavy lifting, aerobic, or fast bicycling. Think only about those physical activities that you did for at least 10 minutes at a time.*** | | | | |
| **No.** | **Questions** | **Choices** | | |  |
| 4.0 | During the ***last 7 days***, on how many days did you do vigorous physical activities? | 1. _______days/week 2. Don’t know/not sure | | |  |
| 4.1 | How much ***total time*** did you usually spend doing ***vigorous physical*** ***activities*** on one of those days? | 1. _______hours/day 2. _______minutes/day 3. Don’t know/not sure | | |  |
| 4.2 | If your pattern of activity varies from day to day, how much ***total time*** did you spend over the last 7 days doing ***vigorous physical activity***? | 1. _______hours/day 2. _______minutes/day 3. _______don’t know/not sure | | |  |
|  | ***Moderate physical activity:***  ***Think about the activities which take moderate physical effort that you did in the last 7 days. Moderate physical activities make your breath somewhat harder than normal and may include carrying light loads, bicycling at a regular pace, or doubles tennis. Do not include walking. Again, think about only those physical activities that you did for at least 10 minutes.*** | | | | |
| **No** | **Questions** | | | **Choices** |  |
| 4.3 | During the last 7 days, on how many days did you do moderate physical activities? | | | 1. _______hours/day 2. _______minutes/day 3. _______don’t know/not sure |  |
| 4.4 | How much ***total time*** did you usually spend doing ***moderate physical activities*** on one of those days? | | | 1. _______hours/day 2. _______minutes/day 3. _______don’t know/not sure |  |
| 4.5 | If your pattern of activity varies from day to day or includes multiple tasks, how much ***total time*** did you spend over the last 7 days doing ***moderate physical activity?*** | | | 1. _______hours/day 2. _______minutes/day 3. _______don’t know/not sure |  |
|  | ***Walking:***  ***Now think about the time you spend walking in the last 7 days. This includes at work and at home, walking to travel from place to place, and any other walking that you might do solely for recreation, sport, exercise or leisure.*** | | | | |
| 4.6 | During the ***last 7 days***, on how many days did you ***walk*** for at least 10 minutes at a time? | | 1. _______days/week 2. _______don’t know/not sure | |  |
| 4.7 | If your pattern of activity varies from day to day or includes multiple tasks, how much ***total time*** did you spend ***walking*** over the last 7 days? | | 1. _______hours/day 2. _______minutes/day 3. _______don’t know/not sure | |  |
| . | ***Sitting:***  ***Finally, think about the time you spent sitting on weekdays during the last 7 days. Include time spent at class, at home, while doing course work, and during leisure time. This may include time spent sitting at a desk, visiting friends, reading, and sitting or lying down to watch television.*** | | | | |
| 4.8 | During the ***last 7 days*** how much ***total time*** did you usually spend sitting on a week day? | | 1. _______hours/weekday 2. _______minutes/weekday 3. _______don’t know | |  |

**Section V: ANTHROPOMETRIC MEASURMENTS**

| Anthropometry | | | | |
| --- | --- | --- | --- | --- |
| No |  | Reading 1 | Reading 2 | Average |
| 1 | Height | __________ | _________ |  |
| 2 | Weight | __________ | _________ |  |
| 3. | Waist circumference | __________ | _________ |  |
| 4. | Hip circumference | __________ | _________ |  |
| 4. | Blood pressure | _________ | _________ |  |

Thank You!!
